# Supplementary figures and images for: Paeoniflorin improves functional recovery through repressing neuroinflammation and facilitating neurogenesis in rat stroke model
Source: PeerJ. 2021 May 28;9:e10921. doi: 10.7717/peerj.10921 (PMC8166241; doi:10.7717/peerj.10921)

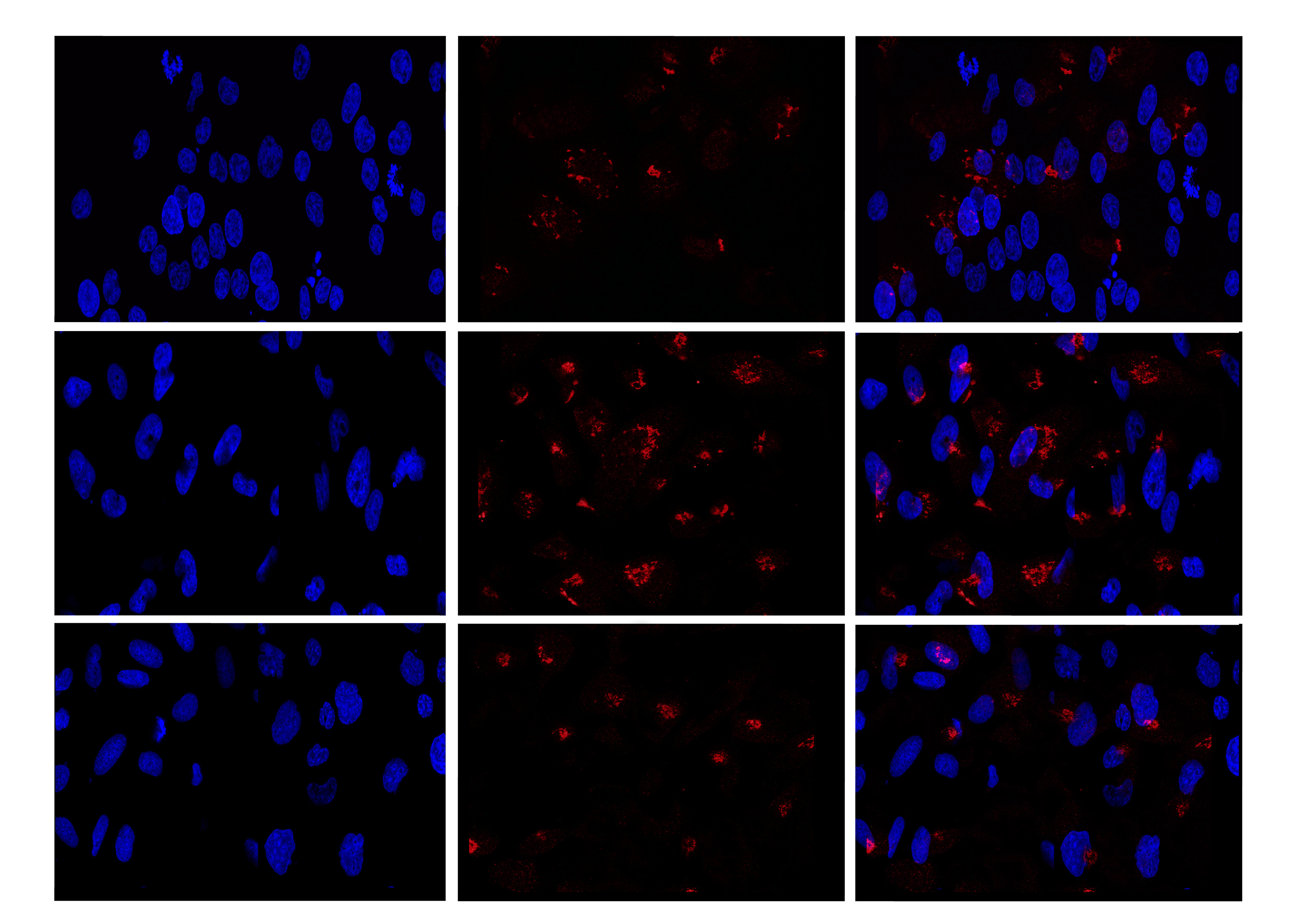

Supplement: Data S1 — Western Blot, immunofluorescence, and qPCR data. [file peerj-09-10921-s001.zip › Raw Data/WBú1⁄4immunofluorescence and qPCR Raw Data/Figure 2A.tif]

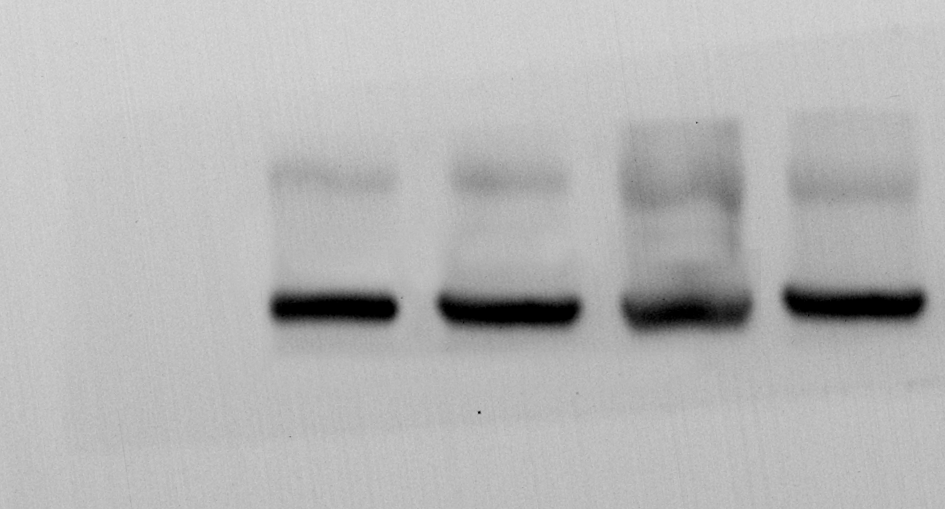

Supplement: Data S1 — Western Blot, immunofluorescence, and qPCR data. [file peerj-09-10921-s001.zip › Raw Data/WBú1⁄4immunofluorescence and qPCR Raw Data/Figure 2B-Actin.tif]

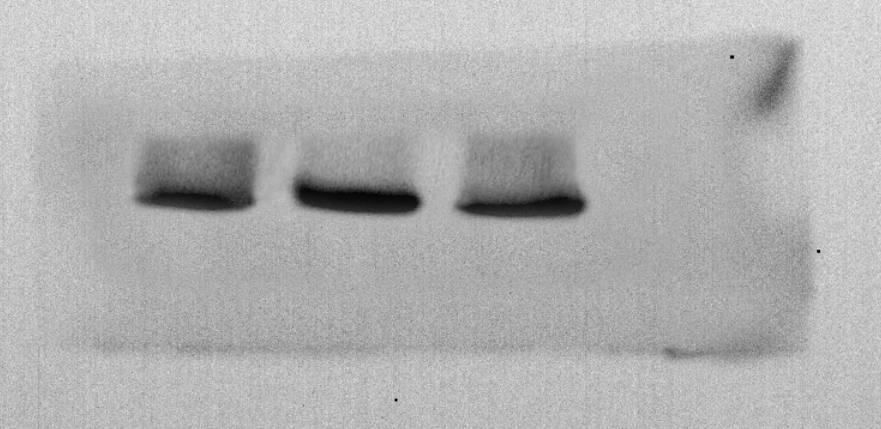

Supplement: Data S1 — Western Blot, immunofluorescence, and qPCR data. [file peerj-09-10921-s001.zip › Raw Data/WBú1⁄4immunofluorescence and qPCR Raw Data/Figure 2B-Iba1.tif]

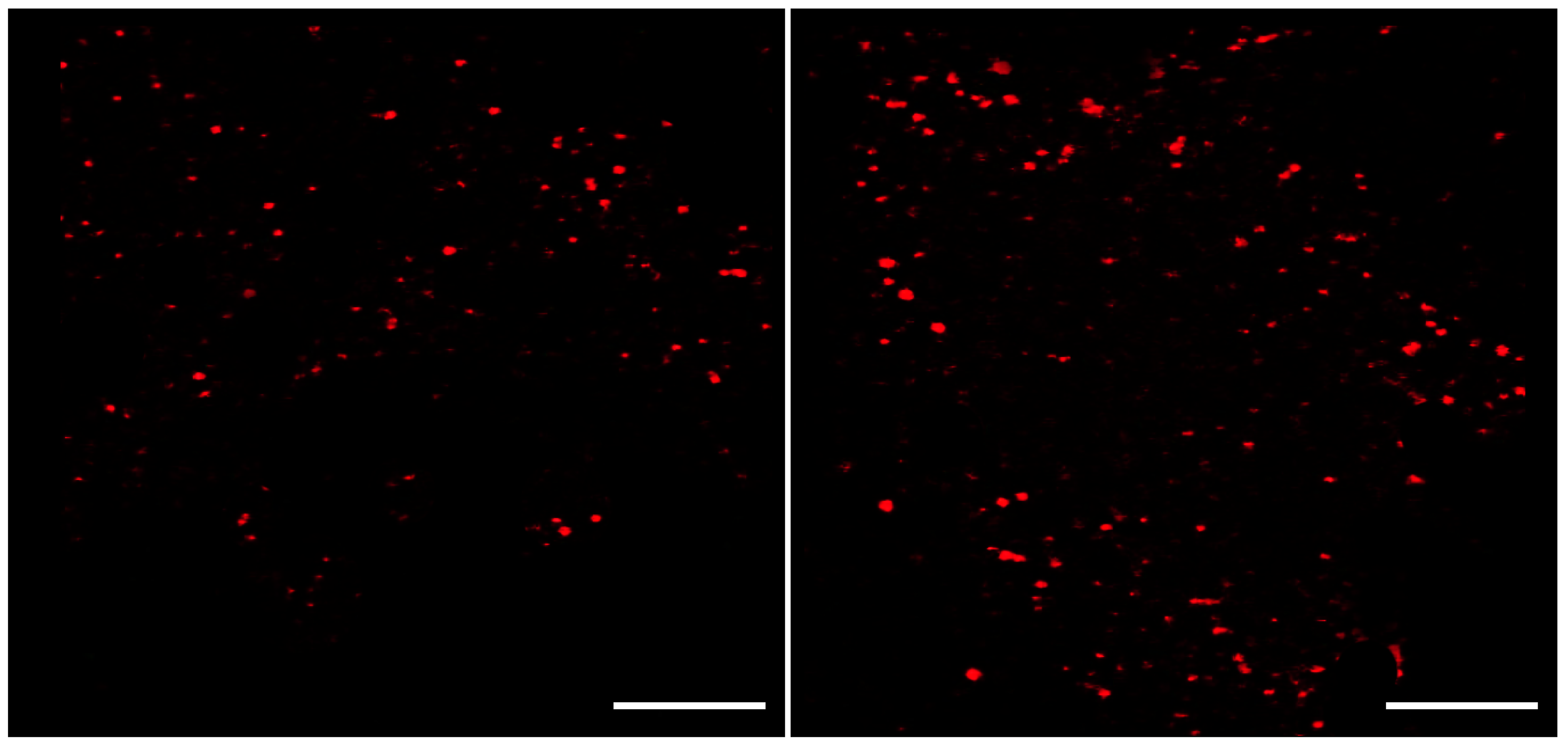

Supplement: Data S1 — Western Blot, immunofluorescence, and qPCR data. [file peerj-09-10921-s001.zip › Raw Data/WBú1⁄4immunofluorescence and qPCR Raw Data/Figure 5A.tif]

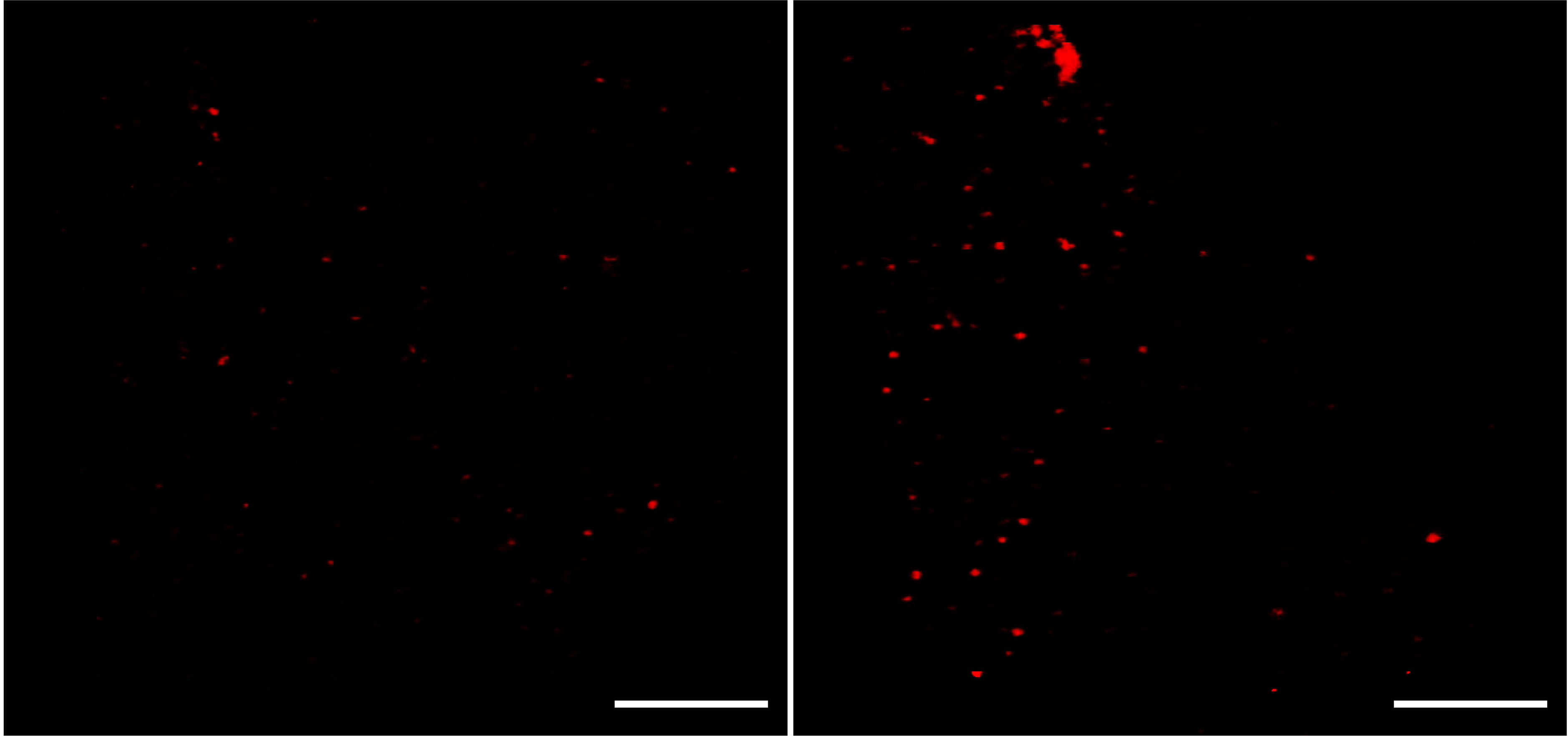

Supplement: Data S1 — Western Blot, immunofluorescence, and qPCR data. [file peerj-09-10921-s001.zip › Raw Data/WBú1⁄4immunofluorescence and qPCR Raw Data/Figure 5B.tif]

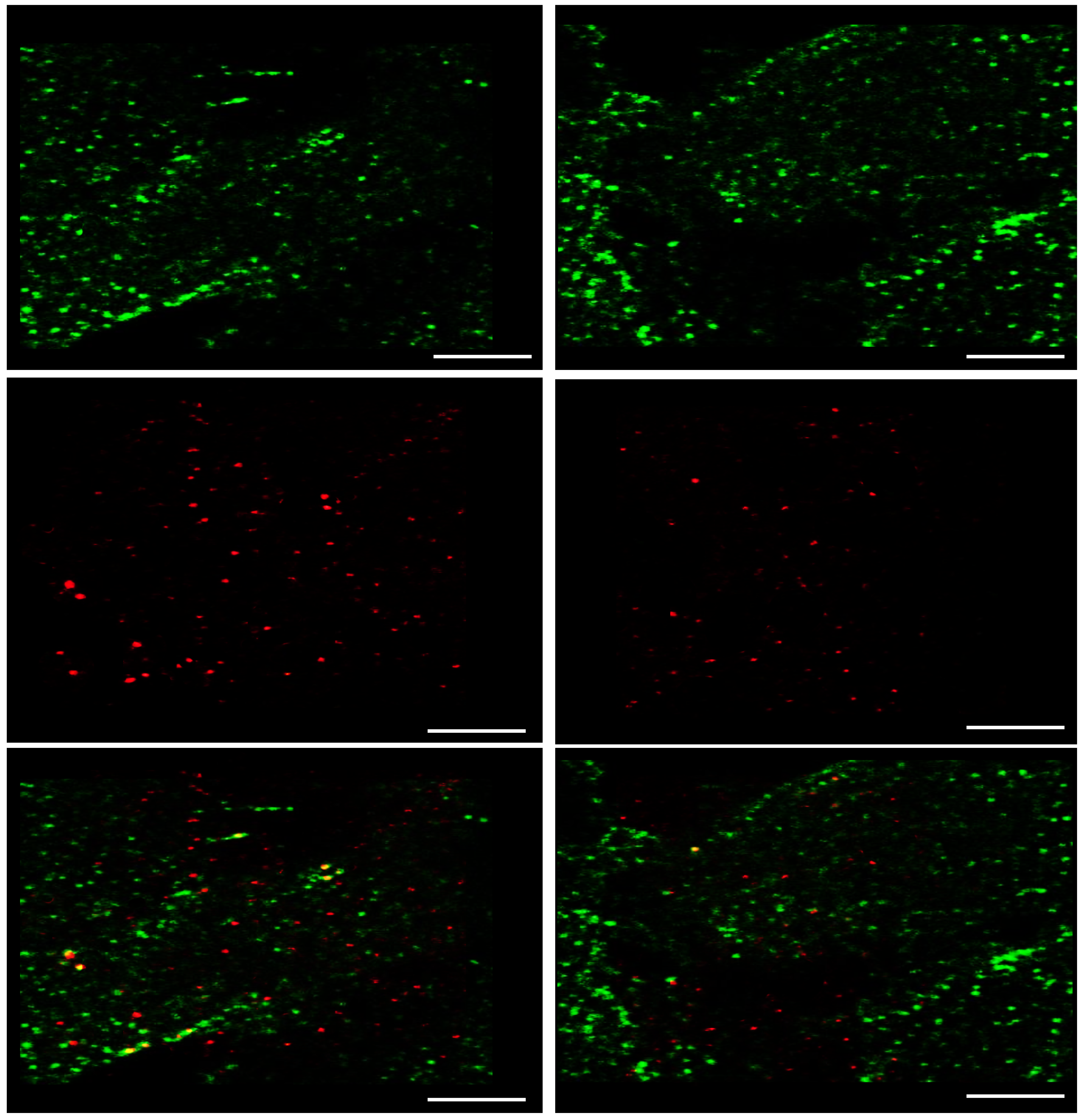

Supplement: Data S1 — Western Blot, immunofluorescence, and qPCR data. [file peerj-09-10921-s001.zip › Raw Data/WBú1⁄4immunofluorescence and qPCR Raw Data/Figure 5C.tif]

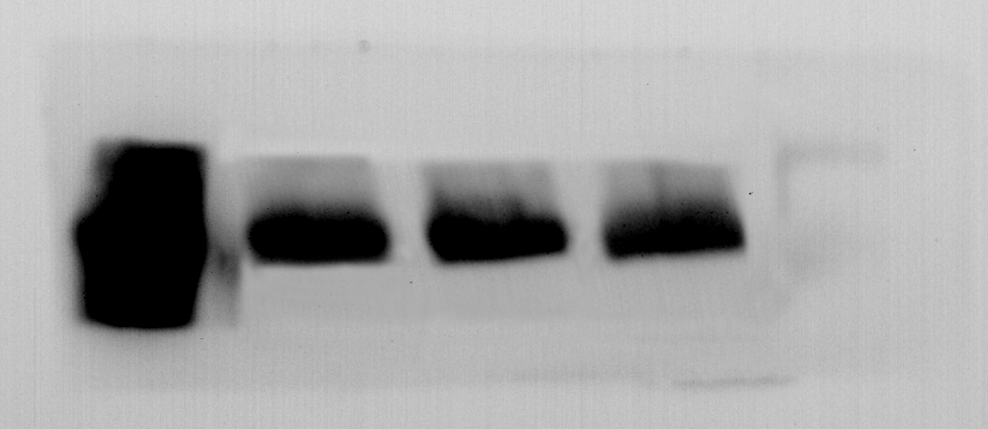

Supplement: Data S1 — Western Blot, immunofluorescence, and qPCR data. [file peerj-09-10921-s001.zip › Raw Data/WBú1⁄4immunofluorescence and qPCR Raw Data/Figure 6A-Actin.tif]

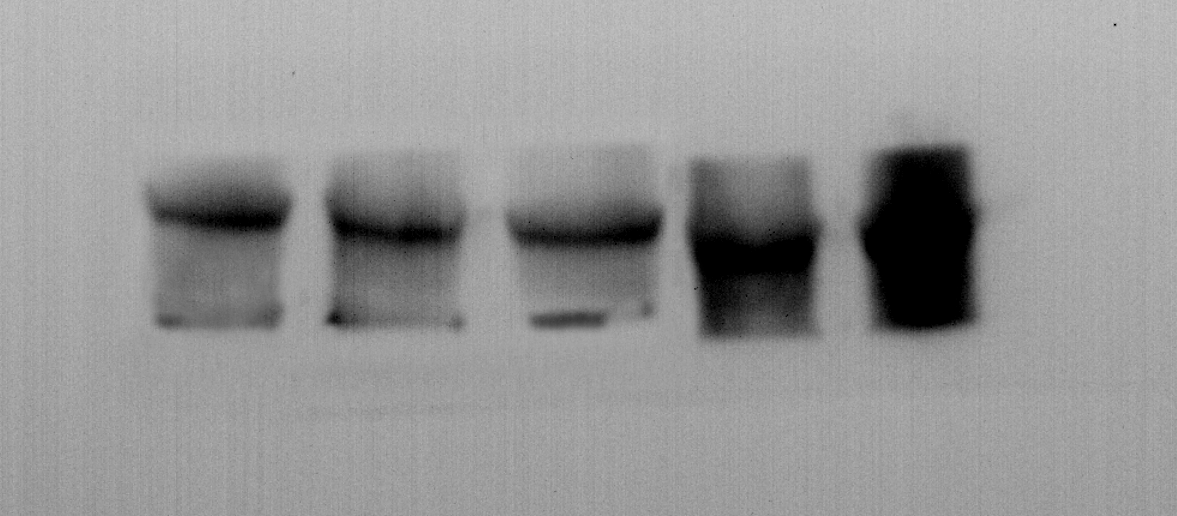

Supplement: Data S1 — Western Blot, immunofluorescence, and qPCR data. [file peerj-09-10921-s001.zip › Raw Data/WBú1⁄4immunofluorescence and qPCR Raw Data/Figure 6A-JNK.tif]

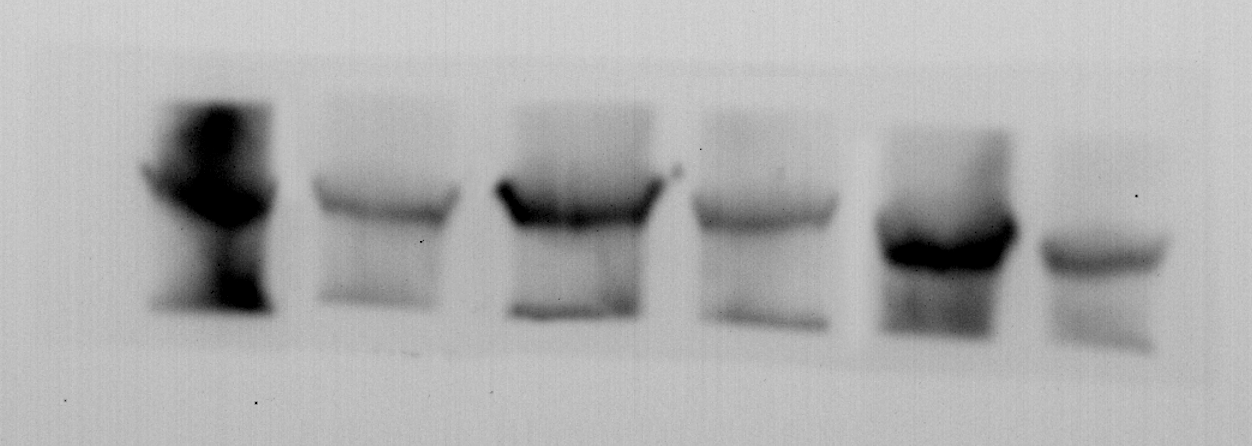

Supplement: Data S1 — Western Blot, immunofluorescence, and qPCR data. [file peerj-09-10921-s001.zip › Raw Data/WBú1⁄4immunofluorescence and qPCR Raw Data/Figure 6A-pJNK.tif]

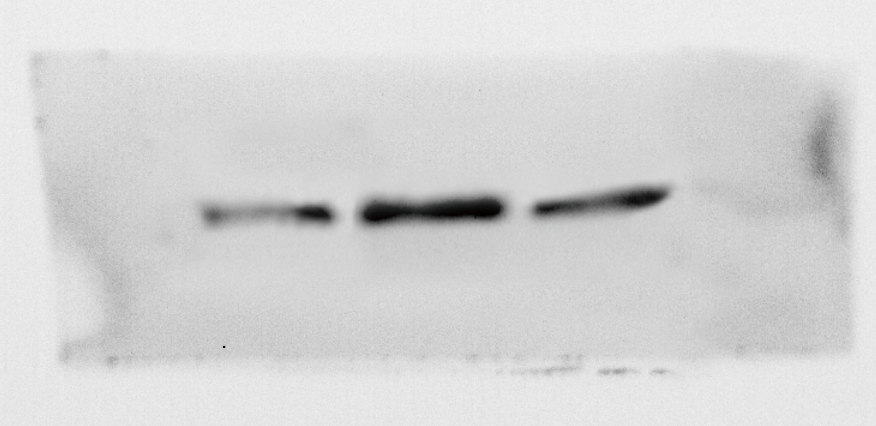

Supplement: Data S1 — Western Blot, immunofluorescence, and qPCR data. [file peerj-09-10921-s001.zip › Raw Data/WBú1⁄4immunofluorescence and qPCR Raw Data/Figure 6C.tif]

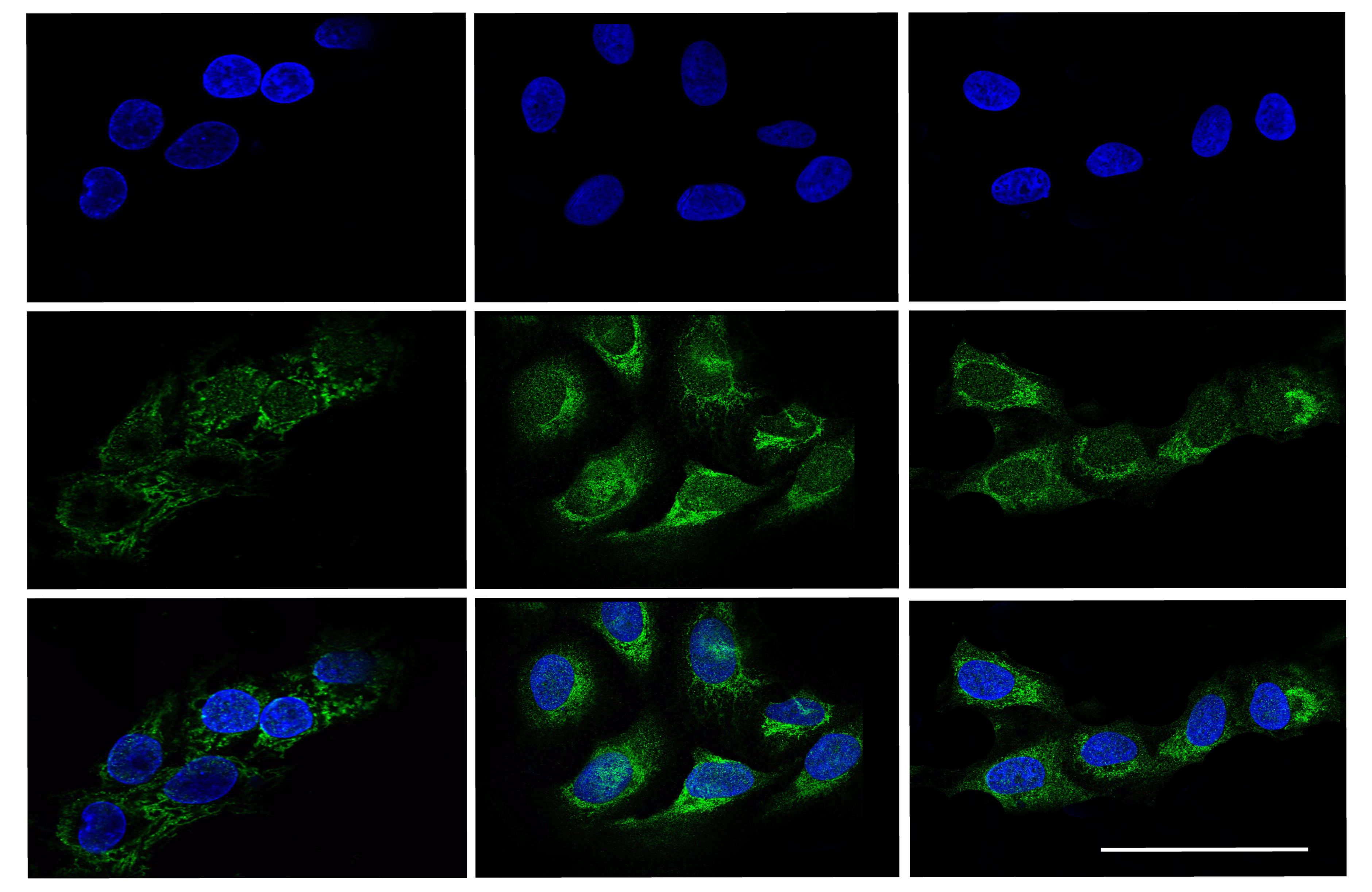

Supplement: Data S1 — Western Blot, immunofluorescence, and qPCR data. [file peerj-09-10921-s001.zip › Raw Data/WBú1⁄4immunofluorescence and qPCR Raw Data/Figure 6E.tif]
